# Recent Advances in the Chemistry and Therapeutic Evaluation of Naturally Occurring and Synthetic Withanolides

**DOI:** 10.3390/molecules27030886

**Published:** 2022-01-28

**Authors:** Amandeep Singh, Asif Raza, Shantu Amin, Chendil Damodaran, Arun K. Sharma

**Affiliations:** 1 Department of Pharmacology, Penn State Cancer Institute, CH72, Penn State College of Medicine, Penn State Milton S. Hershey Medical Center, 500 University Drive, Hershey, PA 17033, USA; asingh8@pennstatehealth.psu.edu (A.S.); mraza@pennstatehealth.psu.edu (A.R.); sga3@psu.edu (S.A.); 2 Department of Pharmaceutical Sciences, Texas AM University, College Station, TX 77842, USA; chendamodar@tamu.edu

**Keywords:** withanolides, withaferin A, anticancer, anti-inflammatory, anti-bacterial, anti-leishmaniasis

## Abstract

Natural products are a major source of biologically active compounds that make promising lead molecules for developing efficacious drug-like molecules. Natural withanolides are found in many flora and fauna, including plants, algae, and corals, that traditionally have shown multiple health benefits and are known for their anti-cancer, anti-inflammatory, anti-bacterial, anti-leishmaniasis, and many other medicinal properties. Structures of these withanolides possess a few reactive sites that can be exploited to design and synthesize more potent and safe analogs. In this review, we discuss the literature evidence related to the medicinal implications, particularly anticancer properties of natural withanolides and their synthetic analogs, and provide perspectives on the translational potential of these promising compounds.

## 1. Introduction

Phytochemicals are a rich resource for small molecule drug discovery and have provided leads for several approved drugs and those entering clinical trials [1]. The development of the first commercial drugs, such as morphine and aspirin, proved the capability of the successful purification and synthesis of drugs from plants [2]. Many of the FDA-approved anti-cancer molecules are natural products or direct synthetic derivatives of natural products, of which plants are the most compelling source [1,3,4]. Although natural products have been extensively used in drug discovery, it is thought that there are still many unexplored plant-based sources used in traditional medicine that could be probed to design and develop new pharmaceuticals in modern medicine [5].

**Figure 1:**
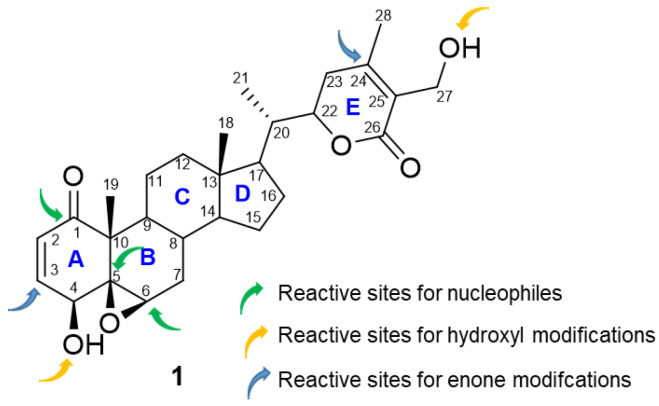
Structure of Withaferin A (WA, **1**) and its reactive sites exploited for structural modifications.

Withanolides belong to a group of naturally occurring C-28 steroid lactones [6]. These compounds are generally highly oxygenated, and these functionalities have led to many structural modifications [7,8,9,10]. The current classification of withanolides constitutes 22 types based on their structural differences. Withanolides, isolated primarily from genera belonging to the plant family Solanaceae, have captured interest mainly due to their diverse structural features and significant biological activities against several diseases [11,12,13,14,15,16,17,18]. *Withania*, a genus of Solanaceae, has been used for over 3000 years as a medicine in Southeast/Southwest Asia in the Ayurvedic system. Ashwagandha (*Withania somnifera*), Indian Ginseng, is the most popular herb commonly used to improve both physical and mental health [19,20]. Traditionally, topical application of the berries and leaves of this plant is used as a remedy for ulcers and tumors [7]. Apart from that, it also showed anti-stress [21,22], anti-inflammatory [23,24,25,26,27], anti-oxidant [24,28,29,30], and anti-depressant [31,32] properties [33,34,35,36]. Withanolides, with 28 carbons of naturally occurring steroids, are the main chemical constituents of the *Withania* genus [37]. Withaferin A (WA) is one of the most active withanolides, and it is mainly responsible for the bioactivity of Ashwagandha [7,10]. WA has shown anti-diabetic, anti-cancer [38,39,40,41,42,43,44,45,46], and anti-angiogenesis [47,48] effects and has attracted the attention of the scientific world to understand its mechanism(s) of action. This is despite the fact that structurally, the three potential reactive sites susceptible to nucleophilic attack, e.g., the unsaturated A-ring at C3, the epoxide structure at position 5, and C-24 in its E-ring (Figure 1) [7,49], have been demonstrated to bind covalently to cysteine residues of proteins, thereby causing loss of activity of the target protein. WA has been shown to exert its anti-cancer effect through the induction of apoptosis in various human cancer cell lines, including breast, prostate, leukemia, colon, pancreatic, renal, head and neck, and many others. WA has been shown to inhibit pro-survival signaling, such as Notch1, MAPK and NF-κB activation, and concomitantly induce pro-apoptotic signaling that results in the growth inhibition of many cancer types [50,51].

This article primarily focuses on the anti-cancer activity of naturally occurring withanolides and their synthetic analogs. Cancer, characterized by the abnormal growth of cells, is the second most fatal disease globally. Several factors, such as tobacco use, over body weight, gene mutation, and hormone and immune conditions, are major causes of the disease [52]. According to WHO, about 14 million new cases are found annually, with 9 million deaths in 2018. Alarmingly by 2030, cancer-related deaths are predicted to increase to about 13.1 million [53]. Although the cause of cancer is related to the unchecked growth of cells, its pathogenesis is extremely complex and involves several interlinked mechanisms. Some of the most studied mechanisms include sustaining proliferative signaling, evading growth suppressors, resisting cell death, enabling replicative immortality, activating invasion and metastasis, inducing angiogenesis, tumor-promoting inflammation, genome instability, and mutation, evading immune destruction, and reprogramming energy metabolism [54,55]. Many anti-cancer agents have been developed, which play an important role in the different steps, such as kinase inhibitors acting on the signal transduction in cells, B-cell lymphoma-2 (Bcl-2) family proteins inhibitors regulating the apoptosis, cell mitosis regulators, epigenetic modifiers, immune regulators, and so on [56,57,58,59]. The Food and Drug Administration (FDA) has approved approximately 150 anti-cancer agents, but a complete cure for cancer remains a challenge. Most of the chemotherapies remain unsuccessful due to acquired resistance. Compounds derived from natural products, such as withanolides, as such or after structural optimizations, may provide effective and safer drug candidates with lower systemic toxicity. Thus, having a high therapeutic index could lead to the development of more efficacious anti-cancer drugs.

## 2. Withanolides as Anti-Cancer Agents

This section provides details of the anti-cancer properties of naturally occurring withanolides and their synthetic analogs with a discussion of their structure–activity relationship (SAR) studies.

### 2.1. Natural Withanolides

Since the identification of the first withanolide, WA (**1**), isolated from the Indian medicinal plant *Withania somnifera* in 1965 [58], a growing number of bioactive withanolides with complex and unique structures have been reported [59,60]. Due to their unique structural skeletons, withanolides display diverse biological activities, including anticancer, anti-microbial, anti-inflammatory, and immunoregulatory activities [60]. Therefore, they are regarded as important origins of lead compounds and potential drug candidates and, thus, over the years, have attracted the interests of chemists and biologists [61]. Withanolides mainly occur in the family of Solanaceae, exemplified by the genera of *Physalis*, *Datura*, and *Withania*.

#### 2.1.1. Physalis

The genus *Physalis* has more than 100 species and is widely spread in tropical and temperate regions [61]. Many species of this genus have been used as traditional medicine worldwide. For example, ‘Ku-Zhi’, a folk medicine of *Physalis angulata* (*P. angulata*), was used to treat impaludism, dermatitis, tracheitis, rheumatism, and hepatitis and was also adopted for similar usage in Mexico, Indonesia, Peru, and Brazil. The fruits, leaves, and calyxes of *P. philadelphica* have been used to treat diabetes, fever, cough, amygdalitis, and gastrointestinal disorders [61]. The medicinal properties of *Physalis* have generated interest in extracting and characterizing compounds that its different species possess and identifying the active withanolides responsible for their medicinal properties.

**Figure 2:**
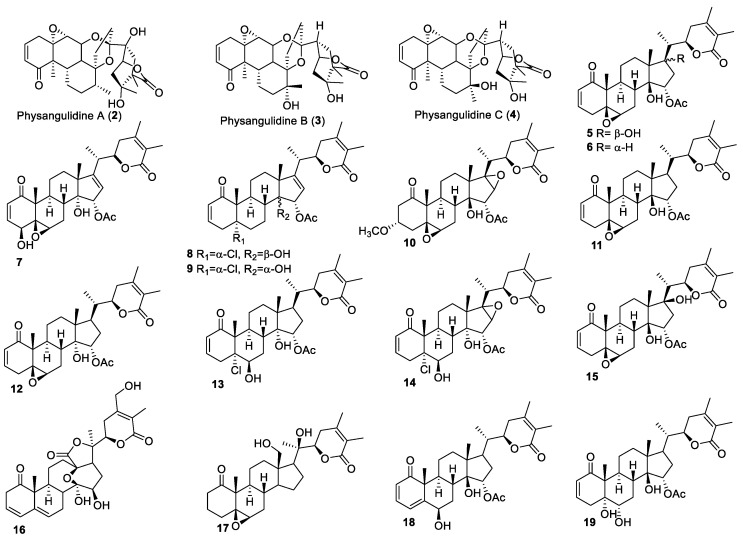
Withanolides isolated from *P. angulata*.

Jhuang Jin and co-workers isolated withanolides **2**–**4** (Figure 2), with an unusual carbon structure, from *P. angulata* L using a bioassay-directed isolation technique [62]. All the three isolated withanolides, Physangulidine A, B, and C, were evaluated for their anti-proliferative activities against prostate cancer cells (DU145) and prostate epithelial cells (RWPE-1). Physangulidine A was found the most effective of the three and showed a GI_50_ (concentration for 50% of maximal inhibition of cell proliferation) value of 3.0 and 2.4 μM against DU145 and RWPE-1 cells, respectively. Physangulidine B and Physangulidine C displayed GI_50_ values in the range of 6.0–6.8 μM against RWPE-1 and DU145 cells. Physangulidine A also showed similar cytotoxicity against colon adenocarcinoma (HT-29) and hematopoietic malignant cells (K562) cells, with a GI_50_ value of 2.73 μM against each cell line as compared to 5-fluorouracil (5-FU) with GI_50_ 5.92 and 30.32 μM, respectively. Notably, Physangulidine A also exhibited relatively lower cytotoxicity against non-malignant 3T3 cells with a GI_50_ value of 4.12 μM compared to 5-FU with GI_50_ 0.28 μM.

Ting Ma and co-workers reported five new biologically active withanolides **5**–**9** (Figure 2) from the plant *P. angulata var* [63]. They tested them, using cisplatin as a reference drug, against three different human cancer cell lines: hepatocellular liver carcinoma (HepG2), breast cancer (MCF-7), and osteosarcoma (MG-63). All the compounds displayed IC_50_ (concentration of a drug that is required for 50% inhibition of cell viability) values in the range of 0.06–6.73 μM, which were comparable to the reference drug. The most active compounds (**5**, **6**, and **7**), having α,β-unsaturation in the ring A, a 5β,6β-epoxy in ring B, and a lactone ring in the side chain, indicated the importance of these functional groups in the withanolides structure for anti-cancer activity. Mechanistically, the most potent compound, compound **5**, arrested cells in the G_2_/M phase and activated caspase-dependent apoptotic pathways [63]. In addition, the induction of apoptosis by **5** in MG-63 cells was associated with the generation of reactive oxygen species (ROS) and the activation of extracellular signal-regulated kinases (ERKs) and c-Jun N-terminal kinases (JNKs). These pathways are deregulated in several cancer types and, therefore, **5** may be a promising compound for further development as a cancer therapeutic.

Another set of new withanolides, **10**–**15** (Figure 2), isolated from *P. angulate*, were screened against human renal carcinoma cells (786-O, A-498, and ACHN), human prostate cancer cells (C4-2B and 22Rvl), and human melanoma cells (A375-S2) [64]. All the compounds exhibited potent anti-proliferative activities against all the tested cell lines, with IC_50_ values in the range of 0.18−7.43 μM. Withanolides with 5β,6β-epoxy (**10**) and 5α-Cl,6β-OH (**13**, **14**) moieties were the most potent against all the tested cancer cell lines. These compounds also showed a substantial inhibitory effect on nitric oxide (NO) production. All the withanolides showed an IC_50_ value in the range of 1.36–5.56 μM, which was lower than hydrocortisone (IC_50_ = 58.79 μM) used as a positive control.

The DNA barcoding technique was used to identify *P. angulate* from which two new withanolides, **16** and **17** (Figure 2), were isolated [65] and evaluated for their anti-cancer activity against Menogaril-resistant mouse leukemia (P388 cells), cervical carcinoma (Hela), and adenocarcinomic human alveolar basal epithelial cells (A549) using the cell viability assay. Compound **16** was found relatively more active than compound **17** and displayed IC_50_ values in the range of 8.00–11 μM. These compounds also induced apoptosis in A549 cells—compound **16** being nearly twice as potent in inducing apoptosis than compound **17**. Furthermore, a set of two new withanolides, **18** and **19** (Figure 2), was isolated from *P. angulata* using dichloromethane extraction, followed by purification with chromatographic techniques [64]. Evaluation of the anti-cancer property of these compounds against A549, HeLa, and PANC-1 cells revealed compound **18** as the most active compound against A549 and human pancreatic cancer (PANC-1) cell lines, with IC_50_ values of 13.47 and 20.23 μM, respectively. Both the compounds were inactive against HeLa cells.

**Figure 3:**
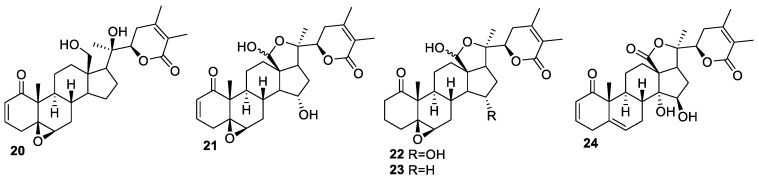
Withanolides isolated from *P. alkekengi*.

Organic extract of another herb, *P. alkekengi* L. var *franchetii* (Mast) Makino, of Solanaceae family [66], found in Asia and Europe, has been shown to exert many medicinal properties, such as anti-inflammatory, anti-oxidant, anti-diabatic, and anti-bacterial [67,68,69]. Five new withanolides, **20**–**24** (Figure 3), were isolated from ethanol extract [70] and were tested for their antiproliferative activities against A549 and K562 cell lines. Compound **20** was the most potent among all the newly isolated withanolides and exhibited IC_50_ values of 4.3 and 2.2 μM against A549 and K562 cells, respectively, comparable to Adriamycin with IC_50_ values of 2.3 and 1.5 μM, respectively, against the two cell lines. A plausible mechanism, evaluated by Western blot analysis as being responsible for the cytotoxicity of compound **20** against A549 cells, involved the regulation of the PI3K/Akt-mTOR signaling pathway.

**Figure 4:**
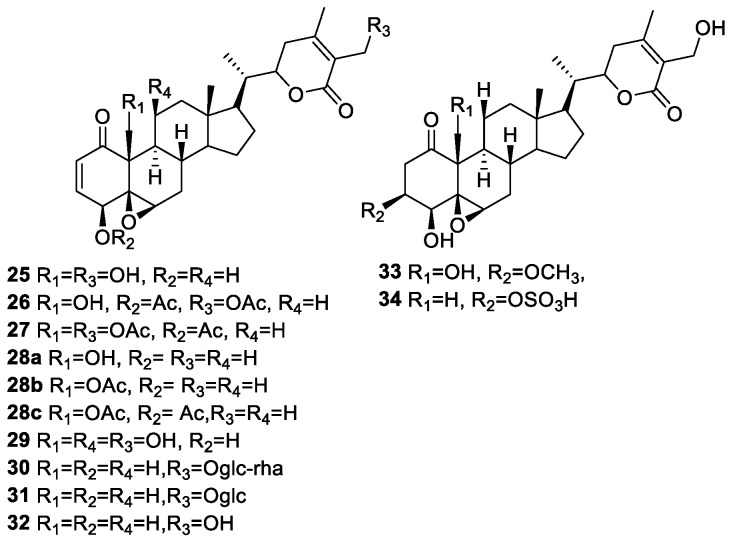
Withanolides isolated from *P. longifolia*.

Huaping Zhang et al. isolated new withanolides (**25**–**34**) (Figure 4) from *P. longifolia* [71] and evaluated them for their cytotoxicity against head and neck squamous cell carcinoma (HNSCC, JMAR, MDA-1986), melanoma (B16F10 and SKMEL-28), and/or normal fetal fibroblast (MRC-5) cells. All the compounds were active against the tested cell lines, with IC_50_ values in the range of 0.067–9.3 μM. Withanolides (**25**–**29**), having a 2-en-1-one in ring A, 5β,6β-epoxy in ring B, and lactone as a side chain, were the most active compounds and demonstrated the importance of these functionalities. Esterification of the OH group at the positions C-4, C-19, and C-27 resulted in an enhanced activity profile; acylated compounds **26** and **28a** exhibited IC_50_ values of less than 1 μM against all the tested cell lines, which are similar to WA (IC_50_ = 0.2–4.0 μM) and less than cisplatin (IC_50_ = 1.0–8.9 μM), used as a positive control. Withanolide glycosides **30** and **31** were less active as compared to WA. The presence of the O-sulfate group in **34** increased the activity profile compared to its parent compound, **32**, against B16F10, JMAR, and MDA-1986 cell lines. Compound **33** was relatively less active, with IC_50_ values in the range of 3.2–10 μM against all the tested cancer cell lines. Compound **26** was the most active among all the isolated new withanolides, having an IC_50_ value of 0.067 μM against B16F10.

**Figure 5:**
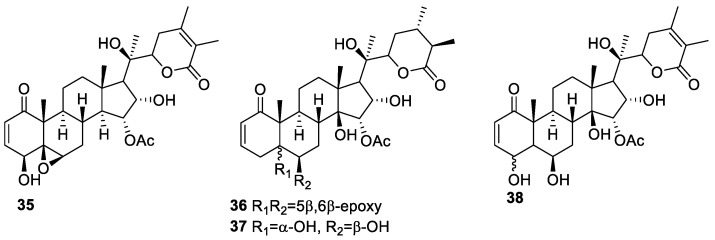
Withanolides isolated from *P. neomaxicana*.

*P. neomexicana* Rydb is commonly found in the United States, primarily in Colorado, New Mexico, Arizona, and Texas. The fruit is called the New Mexico ground cherry and has been used as food by the Acoma, Chiricahua, Laguna, Mescalero, San Felipe, and Rio Grande pueblos of New Mexico [72,73]. Cong-Mei Cao et al. isolated four new withanolides, **35**–**38** (Figure 5), which were evaluated for anti-proliferative activity against MDA-MB-231 and MCF-7 breast cancer cell lines using WA (IC_50_ 0.5 and 1.3 µM, respectively) and withaloglide B (IC_50_ 0.2 and 0.8 μM, respectively) as references compounds. Among all the four new isolated withanolides, compound **36** was most active against MDA-MB-231 and MCF-7 cell lines, with IC_50_ values of 1.7 and 6.3 µM, respectively [74], being less effective than the two reference compounds.

**Figure 6:**
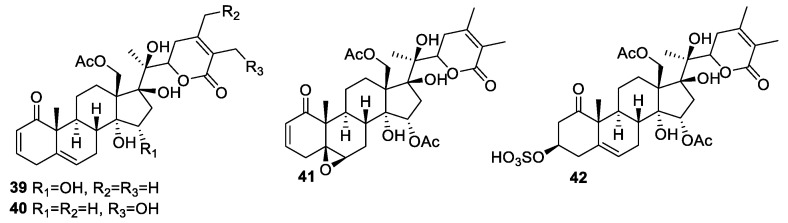
Withanolides isolated from *P. crassifolia*.

*P. crassifolia* Benth extract was found most effective of the 18,000 natural product extracts screened in a high throughput gene-expression assay to treat castration-resistant prostate cancer [75]. Withanolides **39**–**42** (Figure 6) were isolated from *P. crassifolia* and evaluated against five cancer cell lines using doxorubicin as a standard. These compounds were more potent against androgen-sensitive human prostate adenocarcinoma LNCaP (IC_50_ 0.12–3.7 μM) and PC-3M (0.43–5.0 μM) cells compared to the MCF-7 (IC_50_ > 5.0 μM), non-small-cell lung cancer NCI-H460 (IC_50_ > 5.0 μM), and CNS glioma SF-268 (IC_50_ > 5.0 μM) cell lines. The presence of the β-OH group at the C-17 position of the withanolides framework may be responsible for higher potency and selectivity against certain cancer cell lines.

**Figure 7:**
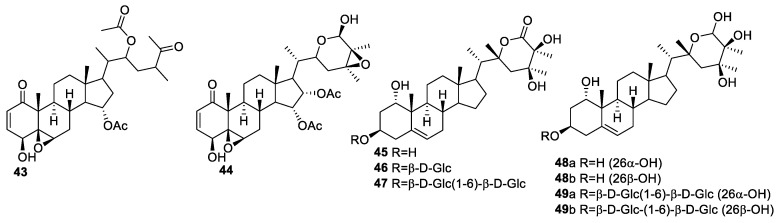
Withanolides isolated from *P. pubescens* L.

*P. pubescens* L, a rich source of withanolides, is used as a medicine to cure sore throat, cough, urethritis, hematuria, and orchitis [75,76] in China. Guiyang Xia et al. isolated withanolides **43**–**49** (Figure 7) from *P. pubescens* L and evaluated them for their anti-proliferative activities against eight human tumor cell lines (C4-2B, 22Rvl, 786-O, A-498, Caki-2, ACHN, A375, and A375-S2) and a human normal hepatic cell line (L02) [77]. Compounds **43** and **44**, with the 4β-hydroxy-2-en-1-one group in ring A and the 5β,6β-epoxy group in ring B, were identified as the most potent against all the tested cancer cell lines, with IC_50_ values in the range of 0.17–5.30 μM. Compounds **45**–**49**, without these functionalities, were inactive, which demonstrated the importance of these functional groups for anticancer activity [77,78,79]. Furthermore, these active compounds were relatively less cytotoxic to the L02 cell line.

**Figure 8:**
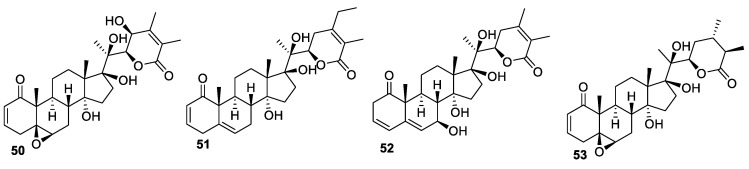
Withanolides isolated from *P. peruviana*.

New withanolides **50**–**53** (Figure 8) were isolated from *P. peruviana* by Ya-Ming Xu and co-workers and were tested against a panel of cancer cell lines consisting of LNCaP, 22Rv1, ACHN, M14 (human melanoma), SKMEL-28 (human melanoma), and normal HFF (human foreskin fibroblast) cells [80]. The compounds displayed stronger activities against LNCaP cells (IC_50_ values of 0.12–2 μM) than other tested cell lines.

#### 2.1.2. *Datura*

The genus *Datura*, which belongs to the family Solanaceae, contains many species indigenous to the American Southwest, including *D. metel*, *D. inoxia*, *D. stramonium*, and *D. wrightii*. The genus originates from the Old World and has a worldwide geographical distribution. Traditionally, Datura extracts are used to treat asthma, earache, headache, and tumors and possess several other medicinal properties, including anesthetic, expectorant, demulcent, intoxicant, hypnotic, and sedative effects. Several withanolides have been extracted from this genus and evaluated for their anti-cancer properties.

**Figure 9:**
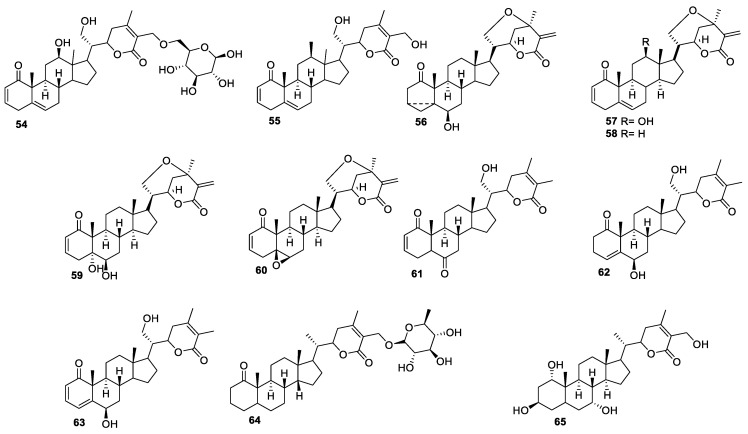
Withanolides isolated from genus *Datura*.

Dinoxin B (12,21-dihydroxy-1-oxowitha-2,5,24-trienolide-27-*O*-β-d-glucopyranoside, **54**) (Figure 9), a new type of withanolide, was isolated from *D. inoxia* leaves using bioassay-guided fractionation [81]. The extracted compound was evaluated for anti-proliferative activity against 21 different cancer cell lines and three normal cell lines. Compound **54** showed cytotoxicity in the sub-micromolar range against several tested cell lines. This activity was unusual for the withanolides with a glucosylated moiety as compared with other withanolide glycosides [10,82]. To determine the effect of the glucosyl group on the activity, compound **55** was generated from **54**. The IC_50_ values for compound **55** against the breast adenocarcinoma viz. T47D, MDA-MB468, and MCF-7 cell lines were 0.99, 0.75, and 0.87 μM, respectively, which are higher than compound **54**, with IC_50_ values of 0.22, 0.58, and 0.61 μM, respectively. In each cell line, compound **54** was relatively more cytotoxic. This is in direct contrast to the bioactivities of other previously reported withanolides, where the presence of a glucose group reduces the cytotoxicity up to >10-fold. For example, the reported IC_50_ values for WA and its glycoside form against MCF-7 cells are 0.6 and 7.9 μM, respectively [10].

The same group further isolated new withanolides from the aerial part of *D. wrightii*, a rich source of C-21 oxygen-substituted withanolides [83]. The five isolated withanolides, **56**–**60** (Figure 9), were evaluated for their cytotoxicity against human glioblastoma (U251 and U87), MDA-1986, and normal MRC-5 cells. The compounds showed IC_50_ values in the range of 0.56 to 3.6 μM against the cancer cells and 3.3 to 5.6 μM against normal fibroblasts. Although these compounds were less potent compared to WA (IC_50_ 0.19–1.1 μM), they were found to be more selective towards cancer cells, with average selectivity of between 4- and 7-fold compared to WA, indicating they might have a higher therapeutic index. However, these compounds should be evaluated for their in vivo efficacy/toxicity to determine if the in vitro results translate in animals and if these compounds have a better selectivity profile at efficacious doses than WA.

Daturanolides **61**–**63** (Figure 9), isolated from the flowers of *D. metel* L, were also evaluated for their cytotoxicity against five human cancer cell lines (HCT116, U87-MG, NCI-H460, BGC823, and HepG2) [84]. However, none of the compounds showed significant activities against the tested cell lines. Two new withanolides, baimantuoluoside H (**64**) and baimantuoluoline K (**65**) (Figure 9), were isolated and identified from the ethyl acetate-soluble fraction of the ethanol extract of *D metel* seeds [1]. The structures of the new compounds were assigned based on 1D, 2D NMR and mass spectrometric techniques. All isolated compounds were evaluated for their antiproliferative activity against human gastric adenocarcinoma (SGC-7901), HepG2, and MCF-7 cells using 5-FU as a positive control. These compounds showed moderate activity against the SGC-7901 cancer cell line, with IC_50_ values of 37.7 and 29.2 μg/mL, respectively, while they were inactive against other tested cell lines. Additionally, these compounds showed immunosuppressive effects, with IC_50_ values of 14.0 and 12.3 μg/mL, which is much lower than the standard drug cyclosporine.

#### 2.1.3. Withania

**Figure 10:**
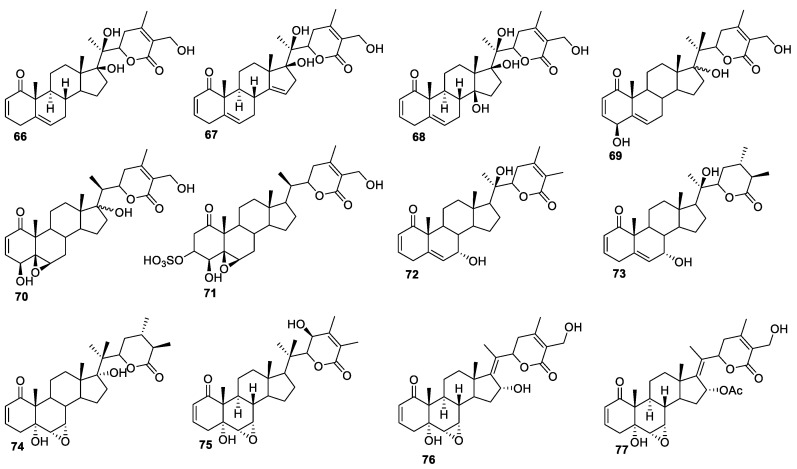
Withanolides isolated from genus *Withania*.

Withanolides are the main phytochemical constituents of the *Withania* genus. Among these, WA has been extensively studied and is shown to be efficacious against several cancers such as glioblastoma, neuroblastoma, multiple myeloma, leukemia, breast, colon, ovarian, and head and neck cancer [85]. Three new withanolides, **66**, **67**, and **68** (Figure 10), were isolated from *Withania coagulans* (*W. coagulans*) [86], which inhibited nitric oxide production in lipopolysaccharide-activated murine macrophage RAW 264.7 cells, with IC_50_ values in the range of 1.9−29.0 μM. The most active compounds, **66** and **67**, showed IC_50_ values of 3.1 and 1.9 μM. These withanolides have also been shown to inhibit tumor necrosis factor-α (TNF-α)-induced nuclear factor-kappa B (NF-κB) activation, with IC_50_ values in the range of 8.8–11.8 μM. Moreover, the compounds isolated from *W. coagulans*, including certain coagulans, coagulanolides, and coagulins isolated from various plant parts, including roots, leaves, and fruits, have been attributed extensive anti-fungal, anti-cytotoxic, anti-diabetic, hypolipidemic, neuroprotective, anti-inflammatory, anti-cancerous, anthelmintic, antioxidant, and wound healing activities [87].

The anti-cancer potential of *W. frutescens*, which is persistently found in Morocco under the name ‘Tirnet’ and traditionally used to treat dysentery [88], has been reported against HeLa 229 cell lines [89]. Laila El Bouzidi and group isolated three new withanolides, **69**–**71** (Figure 10), from Morocco *W. frutescens*, [90], which were evaluated for their cytotoxicity against HT29 cancer cell lines using 5-FU as a reference compound. Compound **71** was the most potent, with IC_50_ value of 1.78 μM, similar to the standard compound 5-FU. Withanolides **69** and **70** were moderately active, with IC_50_ values of 13.18 and 25.13 μM, respectively. The high activity of compound **71** was likely due to its good aqueous solubility and its capability to convert to WA in the cell culture media [91].

Sil Kim and co-workers isolated six new withanolides, **72**–**77** (Figure 10), from *W. somnifera* and screened them against four human cancer cell lines (A549, SK-OV-3, SK-MEL-2, and HCT-15) [92]. Interestingly, all the tested compounds were inactive against A549 cells (IC_50_ values > 10 μM). Withanolides **72** and **73** showed some activities against SK-OV-3 cells, with IC_50_ values of 7.9 and 8.9 μM, respectively. All the compounds were active against all the remaining cancer cell lines, with IC_50_ values ranging from 3.4 to 10 μM.

#### 2.1.4. Other Natural Sources of Withanolides

**Figure 11:**
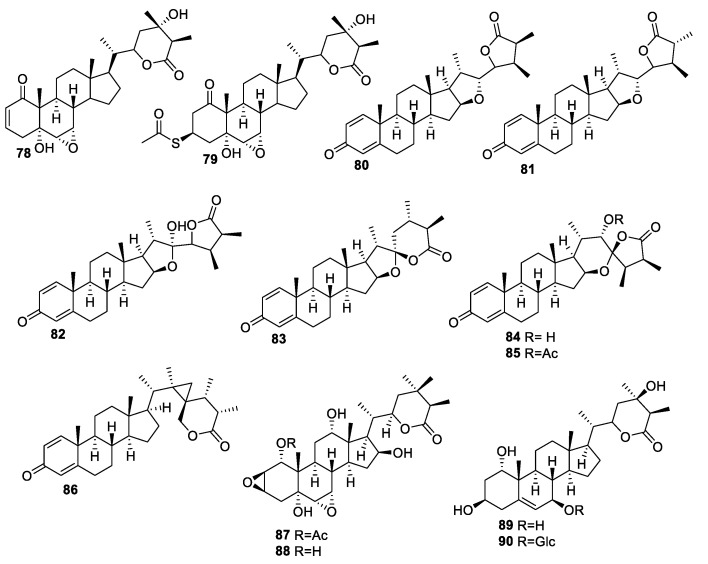
Withanolides isolated from *Dioscorea japonica* **78**–**79**, *Sinularia brassica* **80**–**86**, and *Tacca plantaginea* **87**–**90**.

There are several reports of isolation of withanolides from various other natural sources. Ki Hyun Kim and co-workers extracted two new withanolides, named dioscorolide A (**78**) and dioscorolide B (**79**) (Figure 11), from *Dioscorea japonica* Japanese edible yams, the tropical crops that serve as important staple foods in many parts of the world [93]. The structures of these new compounds were assigned using spectroscopic methods, including 1D and 2D NMR techniques, high-resolution mass spectrometry (HRMS), and chemical methods [94]. The isolated compounds were evaluated against four tumor cell lines (A549, SK-OV-3, SK-MEL-2, and HCT15) and a human healthy cell line (HUVEC) using a sulforhodamine B (SRB) assay. Compound **78** was relatively more potent, displaying IC_50_ values between 6.3–2.9 μM against A549, SK-OV-3, SK-MEL-2, and human colon adenocarcinoma (HCT-15) cell lines compared to **79**, with IC_50_ values in the range of 12.6–25.6 μM. Studies showed that the α,β-unsaturated ketone group in ring A adds to the cytotoxic activity of the withanolides [95,96]. The lower cytotoxicity value of **79** against the SK-OV-3 and SK-MEL-2 cells was attributed to the absence of the α,β-unsaturated double in the ring A. However, the presence of the acetylthio group at C-3 in **79** relatively increases the activity against the A549 and HCT-15 cell lines even though it lacks the 2-en-1-one system in ring A. Both compounds **78** and **79** exhibited lower activity against the normal cell line (HUVEC), with IC_50_ values ranging from 27.1 to 28.8 μM, suggesting selective toxicity towards tumor cells.

Seven novel withanolides, sinubrasolides **80**–**86** (Figure 11), were isolated from the cultured soft coral *Sinularia brassica* [97] and were evaluated for their anti-proliferative activities against P388, lymphoid T carcinoma (MOLT 4), K562, and HT-29 cells. Compound **81** was potent against all the cancer cell lines tested, with ED_50_ values in the range of 4.8 and 9.1 μM, while **84** showed cytotoxicity toward MOLT-4 and HT-29 cells, with ED_50_ values of 9.9 and 7.5 μM, respectively.

Four new withanolides, including one having a sugar moiety, called plantagiolides, were isolated from *Tacca plantaginea*. These new withanolides were evaluated for their TNFα-induced NF-κB transcriptional and cytotoxic activities [98]. The evaluation of the TNFα-induced NF-κB transcriptional activity of compounds **87**–**90** (Figure 11) in human embryonic kidney cells (HEK293T) cells using an NF-κB-luciferase assay showed the new compounds to be inactive, with IC_50_ values greater than 20 μM.

**Figure 12:**
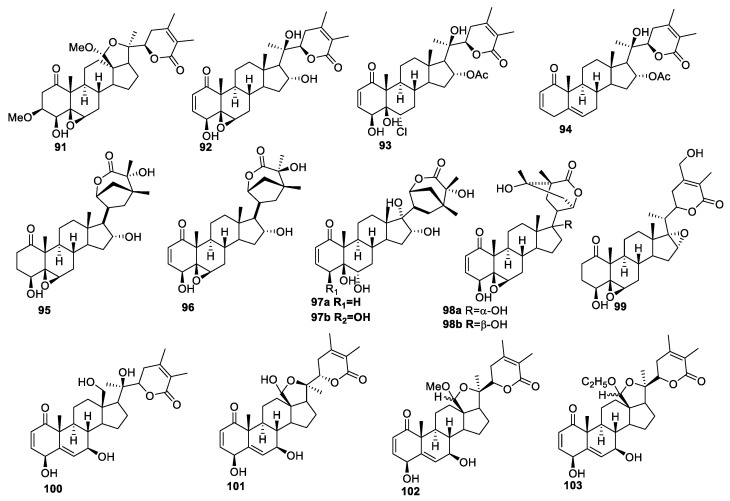
Withanolides isolated from *Acnistus arborescens* (**91**–**94**), *Tubocapsicum anomalum* (**95**–**99**), and *Eriolarynx* and *Deprea* (**100**–**103**).

In order to understand the chemical behavior, Batista and co-workers cultivated Acnistus arborescens and isolated four new withanolides, **91**–**94** (Figure 12), from their leaves using acetone and ethanol extracts [99]. These withanolides were tested for their cytotoxicity against four human cancer cell lines: acute myeloid leukemia (HL-60), human colorectal carcinoma (HCT-116), SF-268, and PANC-1 cell lines. Withanolide **91** was most active against all the tested cancer cell lines, with IC_50_ values in the range of 2.2–4.4 μM. Compound **92** was also active, with IC_50_ values in the range of 2.2–8.0 μM, except for PANC-1 cells, against which it was relatively less effective (IC_50_ value > 10 μM). Compounds **93** and **94** exhibited reduced anti-proliferative potency, probably due to the presence of chlorohydrin in place of the 5β,6β-epoxy group (for **93**) and the lack of OH (at C-4) and the 5β,6β-epoxy group (for **94**) [100].

Ten new withanolides were isolated from *Tubocapsicum anomalum* and were screened for anti-proliferative activities against four human tumor cell lines (HCT-116, HepG2, MCF-7, and A375) using cisplatin as a positive control [101]. Compounds **95**–**99** (Figure 12) were the most potent among all the tested withanolides against all the tested cell lines, with IC_50_ values ranging between 0.24–8.71 μM. SAR studies revealed that the presence of the β-hydroxy group at the C-4 position led to an increase in the activity in **97b**, with IC_50_ values in the range of 3.74–8.10 μM compared to **97a** without the OH having IC_50_ values between 4.42–14.03 μM against all the tested cell lines. Compound **98b**, having β-OH at position C-17, was more active, with IC_50_ values of 1.14, 5.15, and 3.87 μM against Hep-G2, MCF-7, and A375, respectively. On the other hand, compound **98a**, with α-OH at the C-17 position, was less active, with IC_50_ values of 6.07, 14.2, and 13.1 μM against Hep-G2, MCF-7, and A375, respectively.

The genus *Eriolarynx Miers* is found in Peru, Bolivia, and the hot and humid forest of Sierra de Aconquija in northwestern Argentina between the elevation of 1000 and 2000 m. Withanolides isolated from this species have been studied for various biological activities, including anti-inflammatory activity, the induction of quinone reductase, and cytotoxic activity [18,102,103]. Sebastián J. Castro et al. found four new withanolides, **100**–**103** (Figure 12), from Eriolarynx and Deprea, which were evaluated for their anti-cancer activities against A549, HBL-100, HeLa, SW1573, T-47D, and colon adenocarcinoma (WiDr) solid tumor cell lines [104]. Compounds **102** and **103** were identified as the most active compounds, displaying GI_50_ values in the range of 2.4–4.7 μM against all the tested cell lines except for WiDr cells. These two compounds were more effective against A549 cell lines, with GI_50_ = 3.1, 2.8 μM, than the reference drug cisplatin (GI_50_ = 4.9 μM). The four withanolides were also evaluated for their selectivity against the non-cancer cell line BJ-hTERT. Interestingly, all these new compounds showed 2–10-fold selectivity towards the cancer cell lines. The SAR studies revealed that the presence of an acetal group in compounds **101**–**103** instead of hydroxyl (**100**) at the C-18 position increases the activity profiles. Compounds **102** and **103**, with the acetal group, were more potent than compound **101** with the hemiacetal group. The difference in activity between these compounds was associated with their higher lipophilicity, with ClogP values for **101**–**103** being 0.07, 0.54, and 0.93, respectively. In contrast, it is interesting to note that compounds **21**–**24** (Figure 3), with similar acetal groups, led to a loss of activity compared to **20**.

**Figure 13:**
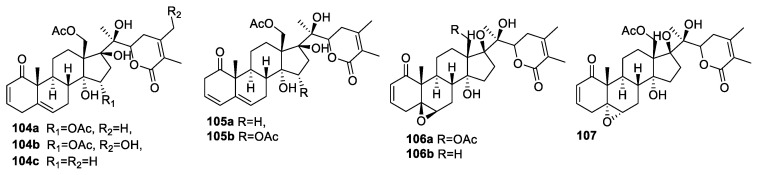
Withanolides isolated from Sonoran desert plants.

Ya-Ming Xu and the group screened an 18,000-member library of Sonoran desert plants [105]. They employed 13,500 fractions in a high-throughput ArrayPlate assay to search for small-molecule natural products that affect the expression pattern of a subset of genes that are known to be involved in prostate cancer [106,107]. The most promising extract provided 15α-acetoxyphysachenolide D (**104a**) [13], 15α-acetoxy-28-hydroxyphysachenolide D (**104b**), 18-acetoxy-17-epi-withanolide K (**104c**), physachenolide D (**105a**), and 15α,18-diacetoxy-17-epi-withanolide K (**105b**) (Figure 13) via gene expression assay-guided fractionation. The compounds inhibited the expression of three androgen-induced genes, KLK2, KLK3, and SPDEF, in DHT-activated LNCaP cells, with IC_50_ values in the range of 0.58–3.58 μM. The most active compound **104c** showed IC_50_ = 0.58, 0.75, and 0.77 μM for KLK2, KLK3, and SPDEF, respectively. Furthermore, the evaluation of cytotoxicity of compounds **104**–**105** against glioma (SF-268), prostate (LNCaP and PC-3), breast (MCF-7), and lung (NCI-H460) cancer cell lines were also done. The results demonstrated compound **104c** to be the most active and selective towards LNCaP and PC-3 cells, with IC_50_ values of 70 and 211 nM, respectively. Many reports have suggested that the presence of the en-1-one moiety in ring A and the 5β,6β-epoxide moiety in ring B increase the activity profile of the withanolides. Among the epoxy derivatives of **104c**, i.e., compounds **106a** and **107** (Figure 13), compound **106a**, with the β-epoxide ring, showed improved activities against the two prostate cancer cell lines, with IC_50_ values of 20 and 90 nM, respectively. In contrast, α-epoxidation (as in **107**) caused a significant loss of cytotoxic activity. Comparing compounds **104**–**107**, containing a 17α side chain and a 17β-hydroxyl group, with WA bearing a 17β side chain, suggested that the orientation of the side chain and/or the presence of the OH group at C-17 may be partly responsible for their significant selectivity to the prostate cancer cell lines, especially LNCaP, compared to other cancer cell lines. In addition, the IC_50_ data of physachenolide C (**106a**) and its 18-deacetoxy analog (**106b**) [108] suggested that the presence of the OAc group at C-18 and/or the absence of the OH group at C-4 in **106a** caused about a 15-fold enhancement in cytotoxic activity for the LNCaP cell line.

### 2.2. Synthetic Analogs of Withanolides

The diverse nature of naturally occurring withanolides presents an equally diverse opportunity to design new analogs with enhanced potency, bioavailability, and reduced toxicity. Therefore, there have been several reports on their modification in the past few decades, including nucleophilic/Micheal addition and hydroxy group modification, leading to a variety of mono-, di-, and tri-substituted withanolides.

#### 2.2.1. Monosubstituted Withanolides

The mono-substituted withanolides primarily involve modifications of the withanolides skeleton at C-27, C-4, and C-3.

##### Modification at the C-27 Position

**Figure 14:**
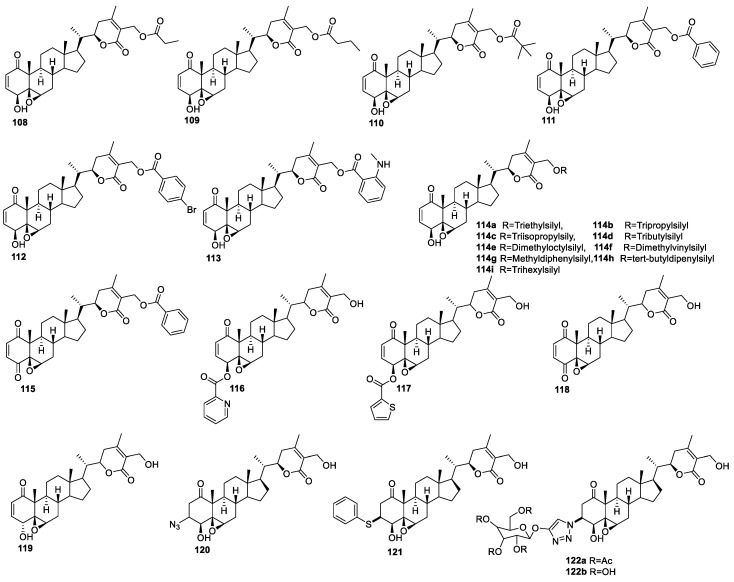
Monosubstituted analogs of WA.

**Figure 15:**
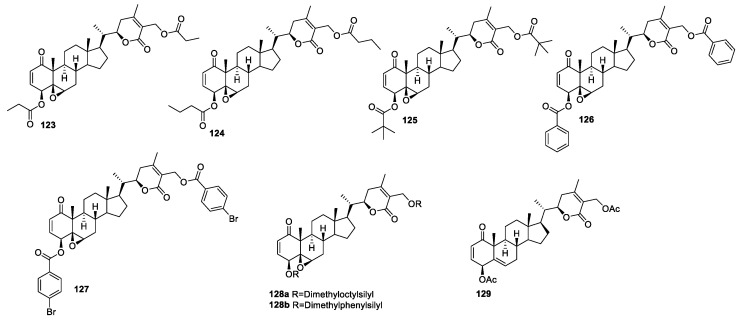
Disubstituted analogs of WA.

Bazzocchi and co-workers designed and synthesized a library of WA analogs (**108**–**113**) (Figure 14) in an attempt to increase its anti-cancer activity profile [46]. The synthesis mainly involved the modifications of the hydroxyl groups, enone system, and 5β,6β-epoxy groups (Figure 15). The compounds were evaluated for their anti-proliferative activity against HeLa, A-549, MCF-7, and non-tumoral (Vero) cell lines. All the synthesized analogs showed good to moderate activity profiles—most of the compounds displayed IC_50_ values ranging from 0.2 to 9.6 μM in all the tested tumor cell lines. A SAR study revealed that compounds having α,β-unsaturated ring A with 5β,6β-epoxide in ring B and the α,β-unsaturated lactone group were the most potent compounds. Compounds with the ester group at the C-27 position were, in general, more active than the non-ester group (WA). Compounds with alkyl esters **108**–**110** showed activities with IC_50_ values of 0.3 to 2.6 μM against all the tested cancer cell lines. The replacement of the alkyl chain on the ester group with the non-substituted benzene ring led to the most promising anti-proliferative compound **111**, with IC_50_ values 0.3–1.6 μM that was attributed to the π–π interaction with the binding site. The addition of electron-withdrawing/donating groups on the benzene **112**–**113** ring decreased the anti-proliferative activity. The most active derivatives (**110** and **111**) induced apoptosis in HeLa cells in a dose- and time-dependent manner, evidenced by chromatin condensation, PS externalization, and caspase-3 activation [109].

The same group further designed and synthesized a library of WA-silyl ether-linked compounds [110] and evaluated them for their in vitro anti-proliferative activity against human epithelial ovarian tumor cell lines, e.g., cisplatin-sensitive (A2780), cisplatin-resistant (A2780/CP70), and non-carcinoma cell lines (ARPE19). Compounds **114a**–**I** (IC_50_ values ranging from 7.3–32 nM) (Figure 14) were more cytotoxic than WA (IC_50_ 32.7 nM) and reference drug carboplatin (IC_50_ 2.6 μM) against the cisplatin-sensitive cell line (A2780). The silyl ether-bearing analog with a dimethyloctyl moiety (**114e**) was 3.2 times more potent (IC_50_ = 10 nM) than WA. Compound **114i**, having a trihexylsilyl moiety at the C-27 position, resulted in complete loss of activity, while compounds **114a**, **114b**, and **144d** were more potent than WA and showed IC_50_ values in the range of 12.8–32.0 nM. SAR studies on a cisplatin-sensitive cell line (A2780) revealed that compounds with heterogeneous alkyl substituents on the silyl ether **114f**–**i** were slightly more cytotoxic than those with the homogeneous silyl ether **114a**–**d**. Regarding the cisplatin-resistant cell line (A2780/CP70), SAR studies of this series of analogs revealed that compounds carrying a heterogeneous alkyl substituent on the silyl ether (**114f**–**i**) displayed similar profiles (IC_50_ 24.9−33.0 nM) to WA, indicating that activity was not greatly influenced by their corresponding silyl ether moiety on the withanolide skeleton.

##### Modifications at the C-4 Position

It has been shown that the conversion of the C-4 hydroxy group of **111** to ketone **115** (Figure 14) reduced the anti-proliferative activity, with IC_50_ values in the range of 1.1–5.4 μM against HeLa, A-549, MCF-7, and non-tumoral (Vero) cell lines [46]. E. M. Kithsiri Wijeratne and co-workers have isolated and semi-synthesized withanolide analogs using microbial and chemical transformation, evaluated them for their cytotoxic activity, and compared them for cytoprotective heat-shock-inducing activity [111]. All the analogs were evaluated for their acute cytotoxicity on human Ewing’s sarcoma cell line CHP-100 using doxorubicin and DMSO as the positive and negative controls. The SAR revealed that the nature of the substituents at the C-4 position had directly affected cytotoxicity. Compounds with a carbonyl group at the C-4 position (**118**, Figure 14), rather than hydroxyl, were inactive compared to WA. The orientation of the OH group in **115** had little effect on the cytotoxicity (IC_50_ 0.93 μM toward CHP-100 cells compared to WA (IC_50_ 0.97 μM)).

Recently, our group was also involved in the modification of the WA structure to develop targeted cancer therapies. This effort has led to several novel analogs with varying degrees of anti-cancer potency (unpublished results). Two of the C4-ester linked compounds reported so far, ASR490 (**116**) and ASR488 (**117**) (Figure 14), have demonstrated promising therapeutic potential against colorectal and bladder cancers, respectively [51,112]. The 2-pyridyl ester-linked ASR490 (**116**) showed the robust growth inhibition of HCT116 and SW620 colon cancer cell lines via the downregulation of the Notch1 signaling pathway. Further, it effectively inhibited tumor growth in control (pCMV/HCT116) and Notch1/HCT116 xenotransplanted mice without any apparent systemic toxicity. The 2-thiophene ester-linked analog ASR488 (**117**) was selectively toxic to bladder cancer cells, while it was relatively non-toxic to normal cells [112]. These reports attest to the therapeutic potential of appropriately designed WA analogs.

##### Modifications at the C-3 Position

Mukherjee et al. [113] synthesized a small library of compounds (**120**–**122**, Figure 14) by regio- and stereo-selective Michael addition to ring A of WA. Ring A of WA was also derivatized by triazoles ring using a Cu(1)-catalyzed 1,3-dipolar cycloaddition reaction with various alkynes. All the synthesized compounds were evaluated for their in vitro anti-proliferative activities against lung, ovary, colon, and prostate cancer cell lines at various concentrations using the SRB assay. Compounds **120**, **121**, and **122** were the most potent of all the synthesized series of compounds against the tested cancer cell lines. SAR studies revealed that a compound with the azide group on ring A (**122**) was significantly more potent than its parent compound, with an IC_50_ value of 0.02–1.9 μM. The activity of the compounds also depended upon the side chain compound **122a**, bearing a galactose moiety, which showed comparable cytotoxicity to WA. Increasing the hydrophilicity by deacetylating **122a** to **122b** resulted in a deleterious effect on growth inhibition. Among the thio group containing analogs, compounds **121** with thiophenol functionality at the C-3 position in ring A was the most potent, with IC_50_ values in the range of 0.13–2.2 μM against the tested cell lines.

#### 2.2.2. Disubstituted Withanolides

The disubstituted withanolides mainly include modification at C-4 and C-27 positions of the withanolide skeleton. In all the cases, generally, the substitution is identical at both the C-4 and C-27 positions because the reaction conditions used for their synthesis lead non-specifically to the acylation of both the hydroxyl groups at C-4 and C-27. Bazzocchi and group synthesized diester derivatives **123**–**129** (Figure 15) using DMAP-promoted acid chloride/acid anhydride coupling with WA [46]. These diesters were marginally less potent than their monoester derivatives, having IC_50_ values in the range of 0.6–4.6 μM against HeLa, A-549, and MCF-7 cells. For example, compound **123** (IC_50_ 1.1–4.1 μM) was slightly less active compared to its monoester derivative **108** (IC_50_ 1.1–2.3 μM) against all the tested cell lines. Disilyl ether containing derivatives of WA were more active than the parent compound WA in human epithelial ovarian tumor cell lines, e.g., cisplatin-resistant (A2780/CP70) and non-carcinoma cell lines (ARPE19). Derivatives **128a** (IC_50_ 1.5 nM) and **128b** (IC_50_ 2.9 nM) were substantially more potent than the parent compound WA (32.2 nM) and exhibited comparable potency to the standard drug carboplatin (2.6 nM) in cisplatin-sensitive A2780 cells. However, these compounds were only slightly more active, with IC_50_ values of 24.9 and 29.0 nM, respectively, than the parent compound WA, having an IC_50_ value of 32.0 nM against cisplatin-resistant A2780/CP70 cells. These compounds were also evaluated against a non-carcinoma cell line (ARPE19) to determine their selectivity; compounds **128a** and **128b** showed good selectivity (selectivity index value > 2) in the non-carcinoma cell line (ARPE19) to A2780 cells. The importance of the epoxy group in the B ring for cytotoxicity has been reported [78,96]. However, the replacement of the epoxy group with a double bond in **129** retained the activity (IC_50_ 0.53 μM) against human Ewing’s sarcoma cell lines (CHP-100 cells), which provides the evidence that the epoxy ring may not be the main contributing factor for cytotoxicity against CHP-100 cells [111].

#### 2.2.3. Trisubstituted Withanolides

**Figure 16:**
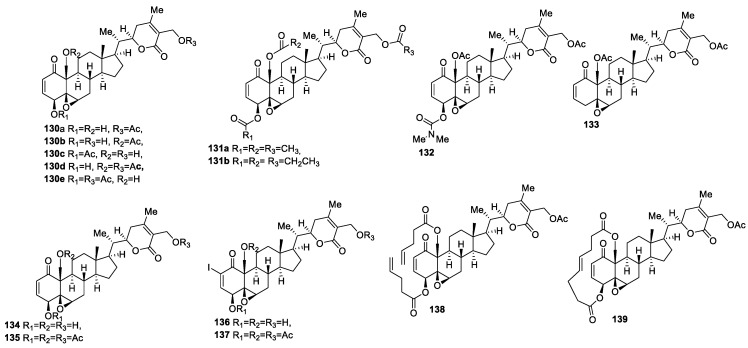
Trisubstituted analogs of WA and related mono- and di-substituted compounds studied for comparison.

There are several reports on the synthesis and biological evaluation of trisubstituted withanolides, bearing substitution, in most cases, at the C-27, C-4, and C-19 positions. Motiwala and co-workers synthesized a series of mono-, di-, and tri-substituted WA analogs, **130**–**137** (Figure 16), and evaluated them for their cytotoxicity against head and neck squamous cell (HNSCC, JMAR), breast cancer (MDA-MB-231), melanoma (SKMEL-28), colon cancer (DRO81-1), and normal fetal lung fibroblast (MRC-5) cells [114]. Di- and tri-acetate analogs **130e** and **131** exhibited enhanced cytotoxic activity compared to monoacetate analogs **130a**, **130b**, and **130c**. Notably, compound **130e** was found to be particularly more cytotoxic toward DRO81-1, with an IC_50_ value of 0.0580 μM. The tripropionylated analog **131** was also active, with IC_50_ values in the range of 0.130–1.00 μM. The increased cytotoxicity of the di- and triacetate analogs of the compound WA could be due to increased lipophilicity, leading to enhanced cell permeability [79,115,116]. Compound **132** showed 3–5 times increased activity, with IC_50_ values of 0.175 and 0.205 μM against breast cancer and melanoma cells, respectively, compared to WA, with IC_50_ = 0.540 and 1.00 μM, while jaborosalactone V diacetate **133** was found to be equipotent to WA, suggesting that the C-4 hydroxyl group is not crucial for activity [8,78]. 2-Iodo analog **136** was inactive, with IC_50_ values of in the range of 5.25 μM–>10 μM compared to its parent compound, **134**, with IC_50_ values of 1.50 μM–5.30 μM against all the tested cell lines. On the other hand, analog **137** showed IC_50_ values in the range of 0.11–0.83 μM against all the tested cancer cell lines, which were equally potent to its precursor compound **135**. Macrocycle **139** exhibited increased potency compared to its acyclic analog **136** across all cell lines tested, with IC_50_ values in the range of 0.205–0.965 μM [117,118]. Most of the active compounds were moderately selective toward cancer cells compared to normal fibroblast cells.

#### 2.2.4. Fused Withanolide Analogs

**Figure 17:**
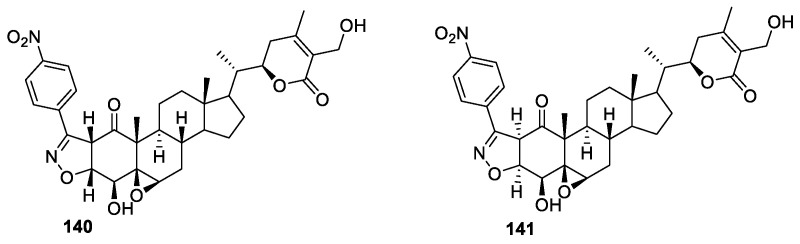
Fused analogs of WA.

The Faheem Rasool group synthesized novel withaferin A-ring-condensed, 2-isoxazoline derivatives [119]. The rationale for designing 2-isoxazoline-fused withanolides was that this moiety has been known as a building block for biologically active molecules and an essential precursor in organic reactions [120]. The synthesized compounds were evaluated for their anti-proliferative activity against MCF-7 and HCT-116 cell lines. All the derivatives showed significant activity against both the tested cancer cell lines. The fused nitro isoxazolines **140** (Figure 17), having a β,β-ring juncture, were found to be more potent against MCF-7 and HCT-116 cell lines, with IC_50_ values 0.70 μM and 1.25 μM, respectively, compared to fused nitro isoxazolines **141** with an α,α-ring juncture, with IC_50_ values of 5.82 and 8.2 μM, respectively.

#### 2.2.5. Generally Adapted Synthetic Approaches

**Figure 18:**
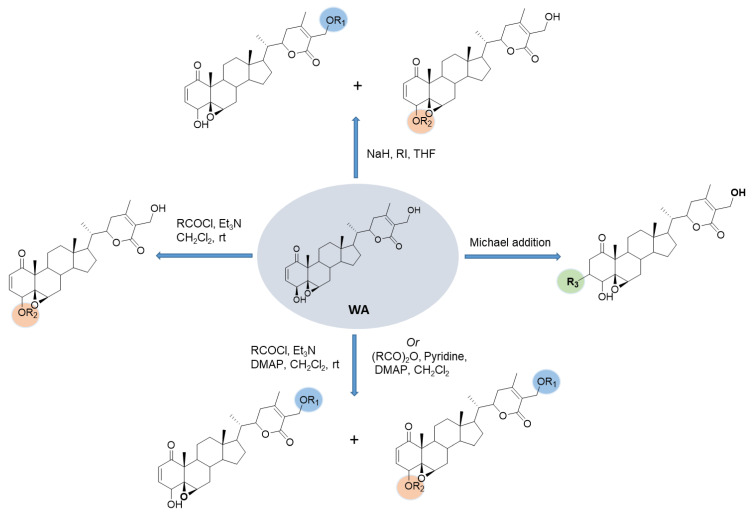
Common synthetic features of withanolide analogs.

Because of the difficulty in synthesizing sterocontrolled withanolide skeletons, most synthetic modifications have been performed starting from a parent withanolide, WA in most cases, using simple synthetic transformations that mainly include the acylation of hydroxy groups at C4 and C27 and a Michael addition to form 3-substituted withanolides (Figure 18). The only interesting synthetic feature that is worth highlighting is the acylation of primary (C-27) vs. secondary (C-4) hydroxyl groups in withanolide structures that can be manipulated based on the reaction conditions used to lead to exclusive or a mixture of mono-primary or secondary hydroxyl group acylations or disubstituted products [114]. For example, the use of acetic anhydride as an acylating agent and pyridine as the base in the presence of DMAP in the acylation of WA [111] led to a mixture of mono-acylated (C-27) and di-acylated (C-4 and C-27) products. A similar pattern was observed for reaction with acid chlorides and trimethylamine and DMAP, which also resulted in a mixture of mono-acylated (C-27) and di-acylated (C-4 and C-27) products [46]. Protection of the primary hydroxyl group was necessary to accomplish the selective acylation of the secondary (C-4) hydroxyl group of WA in these cases. Interestingly, similar reactions of acid chlorides and trimethylamine in the absence of DMAP result in an exclusive formation of the C-4-acylated product, as demonstrated by the selective acylation of the C4-hydroxyl group with excess dimethylcarbamoyl chloride [114] and pyridine-2-carbonyl chloride hydrochloride. However, acylating behavior could also depend on the nature of the acid chloride, as the use of *p*-chlorobenzoyl chloride under similar conditions led to a mixture of compounds with acylation at C-4 and C-27 and multi -OH acylation [114]. Furthermore, sodium-hydride-assisted O-alkylation with methyl iodide led to a mixture of C-4- and C-27-methylated products [46].

## 3. Withanolides as Anti-Inflammatory Agents

Inflammation is a defensive reaction of the body’s immune system against harmful external factors such as bacteria, damaged cells, and irritants [121,122,123]. However, excessive and inappropriate inflammation contributes to various human diseases such as autoimmune disorders, sepsis, diabetes, and even cancer [124,125]. Macrophages play a vital role in the inflammatory response and serve as an essential interface between innate and adaptive immunity [126]. Inflammation can be acute and chronic in acute inflammation; the movement of plasma and leukocytes from the blood into the injured tissue is increased to combat the harmful stimuli, while chronic or prolonged inflammation leads to a gradual change in the form of the cells present at the site of inflammation, such as mononuclear cells, and is characterized by tissue degradation and recovery from the inflammatory process at the same time. This section describes the literature evidence of the anti-inflammatory properties of withanolides.

**Figure 19:**
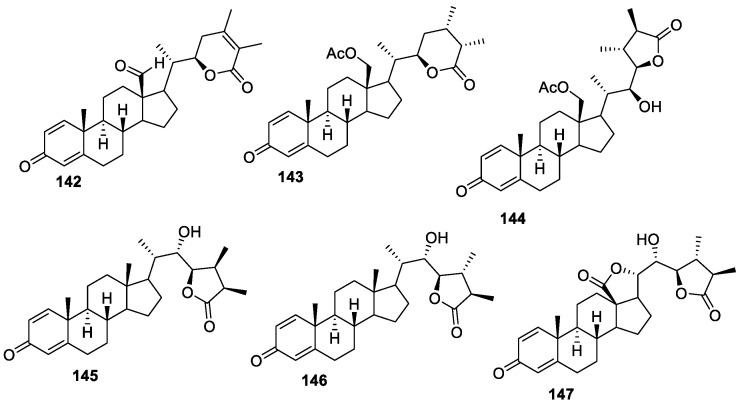
Most potent withanolides from *Minabea* sp.

Minabeolides, the first class of marine withanolides, were isolated from the soft coral *Minabea* sp. by Chih-Hua Chao and group [127] and were tested to inhibit lipopolysaccharide (LPS)-induced pro-inflammatory protein (iNOS and COX-2) expression in RAW264.7 macrophage cells. At a concentration of 10 μM, compounds **142**–**147** (Figure 19) significantly reduced the levels of iNOS to 11.0%, 7.3%, 37.9%, 43.4%, 9.6%, and 45.7%, respectively. However, at this dose, compound **142** showed a decrease of β-actin (66.5% relative to the control group), indicating its cytotoxicity against the tested macrophage cells.

**Figure 20:**
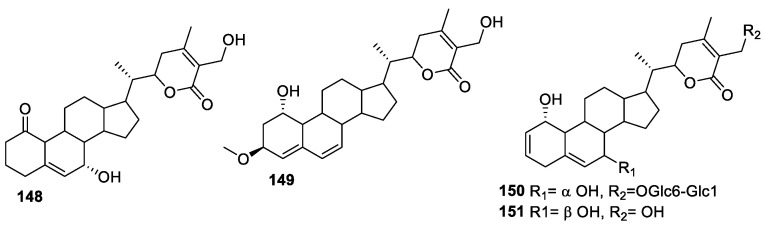
Withanolides isolated from *D. metel* L.

The dry flowers of *D. metel* L, called ‘*Yangjinhua*’, have commonly been used to treat cough, asthma, rheumatism, pain, and convulsions in traditional Chinese medicine for centuries [128]. Withanolides are the main constituents of the flower of *D. metel*, which have been used for the treatment of psoriasis [129]. However, long fluorescence and low yield were the two main disadvantages of dry flowers of *D*. *metel*. Yang and co-workers isolated four withanolides from the leaves of this plant, which has the advantage of high yield [130]. All isolates were identified as major active constituents with inhibitory effects of NO production in LPS-activated RAW 264.7 cells, the murine macrophages [131]. The effect of compounds **148**–**151** (Figure 20) on cell viability was evaluated by the MTT assay to ascertain the absence of cytotoxicity (over 90% cell survival) to macrophage cells at the concentration of 100 μM. Compounds **148** and **151** showed a substantial anti-inflammatory effect, with IC_50_ values of 17.8 μM and 11.6 μM, respectively, compared to L-NMMA (IC_50_ = 13.1 μM) used as a positive control. Compounds **149** and **150** displayed a moderate anti-inflammatory profile, with IC_50_ values of 33.3 and 28.6 μM.

**Figure 21:**
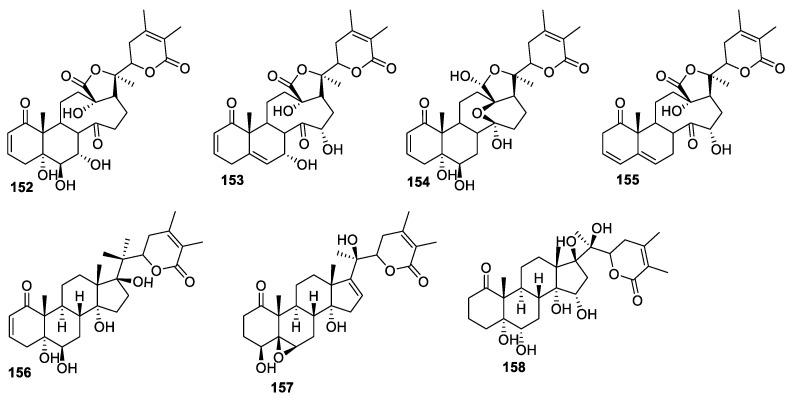
Withanolides isolated from *P. minima*.

*P. minima* is a branched annual shrub that has long been used in traditional medicine in the history of southeastern China with the name ‘*XiaoSuanJiang*’ [8]. Its extracts or infusions have been used to treat many diseases, such as cancer, soreness, pyretic symptoms, and inflammation [132]. Several unique withanolides, such as physalins and withaphysalins, were isolated from this plant [133]. Ru Lin et al. isolated four new withanolides, **152**–**155** (Figure 21), and their structures were confirmed using extensive spectroscopic techniques [102]. These compounds were evaluated for their NO inhibitory effects on LPS-activated RAW 264.7 macrophages to explore their potential anti-inflammatory activity. The MTT assay revealed that all the compounds had no apparent cytotoxic effects on RAW 264.7 macrophages at concentrations of up to 50 μM. Compounds **153** and **155** were moderately inhibitory, with IC_50_ values of 25.34 and 20.81 μM, respectively. Their SAR revealed that 2,5-dien-1-one (**153**) or 3,5-dien-1-one (**155**) moieties in rings A and B are essential for the activity. The absence of this double unsaturation, for example, because of the transformation of 5-ene to 5α,6β-diol (e.g., **152** and **154**), dramatically reduced their NO inhibitory effects. Compounds with s 2,5-dien-1-one moiety were more active than 3,5-dien-1-one for NO inhibition, consistent with the observations made for previously isolated 13,14-seco-withanolides [134]. Furthermore, the introduction of 15α-OH resulted in an obvious decrease in the activity. Three new withanolides, physaperuvin G (**156**), along with physaperuvins I (**157**) and J (**158**) (Figure 21), extracted from the aerial part of *P. peruviana* (Poha), showed moderate to no anti-inflammatory activity, with IC_50_ values in the range of 3–50 μM [135].

**Figure 22:**
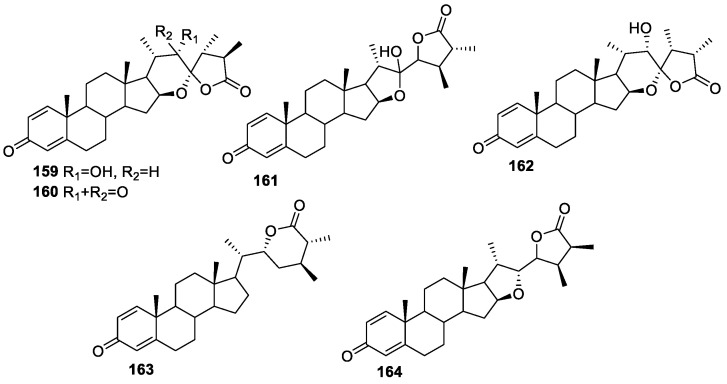
Withanolides isolated from *Sinularia brassica*.

Chiung-Yao Huang and group isolated six new withanolides, **159**–**164** (Figure 22), from *Sinularia brassica* using ethyl acetate extraction [136] and evaluated them for their anti-inflammatory activity on neutrophil pro-inflammatory responses by measuring their ability to suppress fMLP/CB-induced superoxide anion (O_2_^−^) generation and elastase release in human neutrophils. Compound **164** was the most potent, with an IC_50_ value of 3.5 μM against super anion generation and 1.4 μM against elastane release in human neutrophils. On the other hand, compounds **159**–**163** showed a moderate activity profile, with an IC_50_ value greater than 10 μM for both studies.

**Figure 23:**
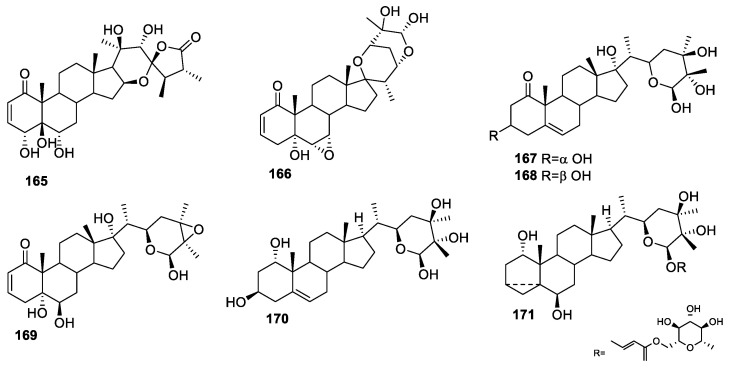
Withanolides isolated from *N. physaloides* and *S. capsicoides*.

Annual herb *Nicandra physaloides* (*N. physaloides; Solanaceae* family) originates in Peru and is also distributed in Yunnan, Guangxi, Guizhou, and other places in China [137]. The whole plant of *N. physaloides* is used as medicine, especially as a sedative, expectorant, antipyretic, and antidote in China [138,139]. Phytochemical studies revealed that *N. physaloides* contained large amounts of withanolides [140,141] and aromatic glycosides [142], with anti-tumor [143], anti-inflammatory [144], and other pharmacological effects. Because of the medicinal property of this herb, Peng Zhang isolated two new withanolides, **165**–**166** (Figure 23), and evaluated them for their inhibitory effects on NO production induced by LPS in a mouse macrophage cell line RAW 264.7, using hydrocortisone and dexamethasone as positive controls. Both the compounds showed low to moderate activity, with IC_50_ values of 58.79 and 7.90 μM [137].

Five new capsisteroids, **167**–**171** (Figure 23), were isolated from *S. capsicoides* by You-Cheng Lin and co-workers [145]. The in vitro pro-inflammatory efficacy of the newly isolated compounds was tested by evaluating the suppression of *N*-formyl-methionyl-leucylphenylalanine/cytochalasin B (fMLF/CB)-induced superoxide anion (O_2_^−^) generation and elastase release in human neutrophils. At a concentration of 30 μM, compounds **168** and **169** were found to inhibit superoxide anion generation (14.8% and 20.9%, respectively). Additionally, compound **170** displayed 42.9% inhibition toward elastase release.

**Figure 24:**
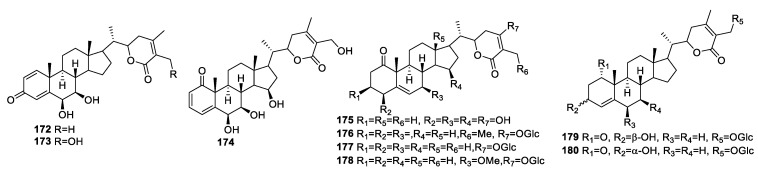
Withanolides isolated from *D. metel* L.

The new undescribed withanolides, daturmetelides **172**–**180** (Figure 24), were isolated from the leaves of *D. metel* L. and were evaluated for their anti-inflammatory activity [146] and mechanism of action. Compounds **172**–**180** were the only active compounds of all the tested compounds, with moderate effect against NO production. Compound **172** showed an IC_50_ value of 13.7 μM against Raw 264.7 cells. From the SAR study, the authors deduced that both the 4*β*-hydroxy-2-en-1-one unit in ring A and a conjugated diene system in rings A and B led to enhanced anti-inflammatory ability. These results further validate that the presence of the 2(3)-en-1-one moiety in ring A plays an essential role in the anti-inflammatory activity, which is consistent with previous reports [135].

## 4. Withanolides as Antibacterial Agents

**Figure 25:**
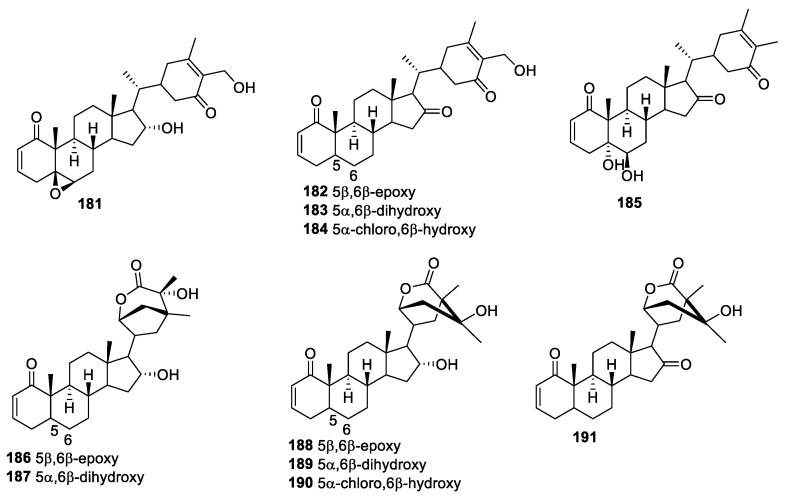
Withanolides isolated from *Nicandra Adans*.

*Nicandra Adans*. (*Solanaceae*), a small genus, has three species, namely, *Nicandra john-tyleriana S. Leiva*, *Pereyra*, and *Nicandra yacheriana S. Leiva* [147,148,149]. Eleven new withanolides, **181**–**191** (Figure 25), were isolated by Nicolas et al. and were evaluated for the antibacterial activity against different strains of Bacillus, Enterococcus, Escherichia, Listeria, Pseudomonas, and Staphylococcus by utilizing a disk diffusion method and bioautography [147]. Compounds **182** and **186** demonstrated effective antibacterial activity against Bacillus. The antibacterial activity of compound **182** was also quantified by direct contact against *B. cereus* BAC1 cells. This compound exerted bactericidal and bacteriostatic effects at 1000 and close to 750 ppm, respectively.

## 5. Withanolides as Anti-Leishmaniasis Agents

**Figure 26:**
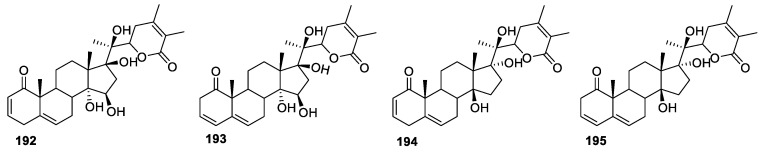
Withanolides isolated from *W. coagulanes*.

In addition to anti-cancer, anti-inflammatory, and anti-bacterial properties, there have been reports of withanolides acting as potential anti-leishmaniasis agents. Leishmaniasis is a parasitic disease occurring in tropical, subtropical, and southern Europe. It is caused by *Leishmania* parasite infection, which is transmitted by the bite of phlebotomine sand flies [150]. Masanori Kuroyanagi and group isolated four new withanolides from the aerial part of *W. coagulanes* and evaluated them for their leishmanicidal activity against *L. major* promastigotes [151]. Compounds **192**–**195** (Figure 26) showed IC_50_ values of 15.9, 50.0, 5.1, and 53.0 μg/mL, respectively, with compound **194** being the only active compound with an IC_50_ value of 5.1 μg/mL.

## 6. Conclusions

Plants are a rich source of naturally occurring small molecules of medicinal importance that make valuable leads for drug discovery. For example, withanolides are naturally occurring C-28 steroid lactones, which have shown tremendous potential as preventive/therapeutic agents for cancer and several other diseases (Table 1). In addition, these compounds are rich in oxygenated functions that provide ample opportunity for structural modifications in order to design more efficacious drug-like molecules. Most of the withanolides have been extracted from the *Solanaceae* family. Traditionally, leaves and barriers of the Ashwagangha (*Withania somnifera*) plant have been used as a remedy for treating several health conditions. Among withanolides, WA is the most active compound that is mainly responsible for the biological activity of Ashwagandha.

Naturally occurring withanolides have shown a variety of biological activities. In addition to the anti-cancer, anti-inflammatory, antibacterial, and anti-leishmaniasis activities described in this article, there is ample evidence of the use of natural withanolides in treating depression, fatigue, and insomnia. Furthermore, they inhibit perimenopausal symptoms, improve sperm viability and mobility, and help in back and neck pain syndromes and arthritis. The powdered plant is also a rich source of iron and stimulates the cells of the immune system. In addition to having such diverse biological activities, natural withanolides provide many reactive sites for their structure modification, which can be used to develop more potent and high-yield compounds. Notably, the structure of WA, one of the most active withanolides, has been extensively modified to improve its anti-cancer potential. The modifications are mainly carried out on the hydroxyl group at the C-4, C-27 positions and on the enone system at the C-3 position. These synthetic analogs showed a better activity profile than the parent compound WA against HeLa, A-549, and MCF-7 cell lines [46]. Silyl ether linkage of WA also led to an increase in potency compared to WA and standard drug carboplatin against the cisplatin-sensitive A2780 cell line. Silyl ether analogs, having a hydroxyl group at the C-4 position (**111**), were more potent than analogs having the ketone group at this position (**115**), indicating that the hydroxyl group may be contributing to the activity in this case [110]. Furthermore, modification on the enone system at the C-3 position also increased the potency of the compound. Analog **120**, having the azide group at C-3, was more potent against lung, ovary, colon, and prostate cancer cell lines than WA [113]. In addition, acetate derivatives of WA were also more active than WA—di- and tri- acetate analogs **130e** and **131** were shown to be relatively more potent, with IC_50_ values ranging 0.11 μM to 0.65 μM compared to monoacetate analogs **130a**, **130b**, and **130c**, having IC_50_ values of 0.415–3.06 μM against head and neck squamous cell (HNSCC, JMAR), breast cancer cells (MDA-MB-231), melanoma (SKMEL-28) and colon cancer (DRO81-1) cells [114]. Among the A-ring-fused isoxazolines, WA analogs, the compounds having a nitro group and β,β ring juncture (e.g., **138**), were shown to be more active than the α,α ring juncture (e.g., **139)** against MCF-7 and HCT-116 cell lines [119]. A recent report from our group indicates that C4-acetylated analog ASR490 (**116**) of WA was significantly more potent than WA in inducing apoptosis in cancer cells. ASR490 effectively inhibited the growth of HCT116 and SW620 colon cancer cell lines by downregulating Notch1 signaling [51]. In addition, ASR490 inhibited the tumor growth in control (pCMV/HCT116) and Notch1/HCT116 xenotransplanted mice without any apparent systemic toxicity. Another C4-acetylated compound, ASR488 (**117**), inhibited the growth of bladder cancer cells while sparing normal cells. Overall, a careful survey of the literature reports suggests that in most cases, the presence of α,β-unsaturation in the ring A modification of the hydroxyl groups at the C-4 and C-27 positions and the β,β ring juncture in the ring-A-fused compounds led to an enhancement in potency.

Thus, based on the SAR of withanolide analogs, the following correlations can be derived: (i) the presence of α,β-unsaturation in ring A is critical for their biological activity, and the absence of a double bond in ring A reduces the activity profiles of the analogs [93]. (ii) Acylation on C-4, C-19, and C-27 results in increased activity against various cancer cell lines [71]. Withanolides isolated from *D. wrightii*, with oxygenation at the C-21 position, exhibited reduced anti-cancer potential compared to WA [83]. (iii) Withanolides having the β-hydroxyl group at C-4 and the 5β,6β-epoxy group in ring B have been shown to be more potent compounds compared to those that are without these functionalities, and (iv) withanolides having the acetal group instead of the hydroxyl group at the C-18 position prove to be the more active compounds as these compounds have high lipophilicity [105].

Most of the WA modifications have been made through the ester bond at the C-4 and C-27 positions. Although many of these derivatives have been found significantly more potent than WA, the ester bonds are relatively unstable and can efficiently cleave in the body, releasing WA. The stability of the analogs can be increased via modification through the amide or triazole linkage. However, this would be challenging, synthetically starting with WA or other withanolide structures. Further, rational modifications of natural withanolides could provide safe and more target-specific compounds for individual diseases. Most of the structural modifications reported thus far are performed starting from withanolides, mostly from WA. This limits the diverse nature of compounds that can be generated. Although these modifications have already led to the discovery of several potent analogs, such structures can be better diversified to achieve potency and specificity towards particular molecular targets by developing synthetic techniques that could include the construction of a steroid skeleton. However, the challenge is to perform multi-step stereocontrolled syntheses of withanolide scaffolds to prepare rationally designed analogs that may not be accomplished by starting from withanolide structures. The synthesis of withaferin A, although multi-step and cumbersome, has been reported [152] and can be exploited as such or with improvements to generate more diverse novel analogs.

Apart from synthetic challenges, another hurdle in identifying potentially translational natural and synthetic withanolides, which have been shown to have varying degrees of activity against different cancer cell lines, is the lack of detailed studies on their efficacy and toxicity. Most of the studies report only in vitro cell viability data against different cancer cell lines, and many of them are not even compared against normal cells. In addition, studies are independently conducted in different labs using different assays, making it challenging to correlate which compounds would be the best for future development. Future studies could identify the top 20–25 most potent compounds, as reported, which can be compared under similar assay conditions and cancer cell lines and against normal cell lines to identify the best of the lot, with a high therapeutic index that could be studied in further pre-clinical in vivo models and, if found worthy, could eventually be developed clinically or serve as lead compounds for further optimization. Prioritizing promising synthetic analogs is also essential to reduce our dependence on natural sources, the production of which can be hampered in the future due to several factors, including extreme weather conditions, batch-to-batch consistency due to both the climate and the soil conditions, and lack of enough space for production due to the increasing world population.

Plants sources of withanolides, particularly the ancient medicinal herb Ashwagandha, have been traditionally used for several maladies. Although the anti-cancer properties of withanolides are well documented, there is also evidence, although insufficient, that Ashwagandha can reduce cholesterol levels, blood sugar levels, anxiety, and stress; improve fertility and testosterone in men, sperm quality, brain function, and sleep quality; and help fight depression and treat hypothyroidism. This information provides ample opportunity to test both natural and synthetically modified withanolides against such health conditions.

In summary, naturally occurring withanolides have shown great promise as pleiotropic therapeutic compounds in both in vitro and in vivo models and make valuable lead molecules for developing more potent and target-specific drug-like compounds. The derivatives developed and studied so far indicate that appropriately designed WA analogs could lead to promising drug candidates for various diseases, particularly cancer, in the near future and thus warrant further development.

## Figures and Tables

**Table 1:** Source and activity of natural withanolides.

Genus	Species	Scaffold	Activity Against	References
*Acnistus*	*arborescens*	Withanolides **91**–**94** (Figure 12)	Leukemia, colon, adenocarcinoma, glioblastoma, and pancreas carcinoma	[99]
*Datura*	*inoxia*	Dinoxin B **54** (Figure 9)	Breast, lung, colon, liver, melanoma, and ovarian cancer	[81]
	*metel L*	Daturanolides **61**–**63** (Figure 9)	Colon, lung, glioma, liver, and human gastric cancer	[84]
		Withanolides **148**–**151** (Figure 20)	Inflammation	[131]
		Daturmetelides **172**–**180** (Figure 24)	Inflammation	[146]
	*wrightii*	Withanolides **56**–**60** (Figure 9)	Human glioblastoma, head and neck squamous cell carcinoma	[83]
*Deprea*	*subtriflora*	Withanolides **100**–**103** (Figure 12)	Lung, cervical, breast, and colon cancer	[104]
*Dioscorea*	*japonica*	Dioscorolide A **78** and dioscorolide B **79** (Figure 11)	Lung, ovarian, melanoma, and colon cancer	[94]
*Eriolarynx*	*iochromoides*	Withanolides **100**–**103** (Figure 12)	Lung, cervical, breast, and colon cancer	[104]
*Morocco*	*W. frutescens*	Withanolides **69**–**71** (Figure 10)	Colon adenocarcinoma	[90]
*Nicandra*	*physaloides*	Withanolides **165**–**166** (Figure 23)	Inflammation	[137]
	*john-tyleriana S. Leiva*, *Pereyra* and *yacheriana S. Leiva*	Withanolides **181**–**191** (Figure 25)	Bacteria	[147,148,149]
*Paraminabea*	*acronocephala* (Soft coral)	Paraminabeolides **142**–**147** (Figure 19)	Inflammation	[127]
*Physalis*	*angulate*	Withanolides **16** and **17** (Figure 2)	Prostate, renal, and melanoma cancer	[65]
	*angulata var*	Withanolides **5**–**9** (Figure 2)	Breast and liver cancer	[63]
	*crassifolia*	Withanolids **39**–**42** (Figure 6)	Prostate adenocarcinoma, breast adenocarcinoma, non-small-cell lung cancer, and CNS glioma	[75]
	*longifolia*	Withanolides **25**–**34** (Figure 4)	Head and neck squamous cell carcinoma and melanoma	[71]
	*minima Linn. var. indica*	Withanolides **152**–**155** (Figure 21)	Inflammation	[102]
	*neomexicana Rydb*	Withanolides **35**–**38** (Figure 5)	Breast cancer	[74]
	*peruviana*	Withanolides **50**–**53** (Figure 8)	Androgen-sensitive and -resistant human prostate adenocarcinoma, human renal adenocarcinoma, and human melanoma	[80]
	*pubescens L*	Withanolides **43**–**49** (Figure 7)	Prostate cancer, renal carcinoma, and melanoma	[77]
*Sinularia*	*brassica* (Soft coral)	Sinubrasolides **80**–**86** (Figure 11)	Murine pre-B-cell lymphoma, acute lymphoblastic leukemia, myelogenous leukemia, and colon cancer	[97]
		Withanolides **159**–**164** (Figure 22)	Inflammation	[136]
*Solanum*	*capsicoide*	Capsisteroids **167**–**171** (Figure 23)	Inflammation	[145]
*Tacca*	*plantaginea*	Sinubrasolides **87**–**90** (Figure 11)	Liver carcinoma	[98]
*Withania*	*coagulans*	Dioscorolide A **78** and dioscorolide B **79** (Figure 11)	Inhibition of TNF-α-induced NF-κB activation in human embryonic kidney cells	[93]
		Compounds **192**–**195** (Figure 26)	Leishmaniasis	[151]
	*somnifera*	Withanolides **72**–**77** (Figure 10)	Breast, lung, colon, liver, prostate, oral, cervical, pancreatic, and ovarian cancer	[92]

## Data Availability

Not applicable.

## Abbreviations

WA, withaferin A; WN, withanolids; MAPK, mitogen-activated protein kinase; NF-κB, nuclear factor ‘kappa-light-chain-enhancer’ of activated B-cell; Bcl-2, B-cell lymphoma-2; GI_50_, concentration for 50% of maximal inhibition of cell proliferation; 5-FU, 5-flourouracil; IC_50_, concentration of a drug that is required for 50% inhibition; ROS, reactive oxygen species; ERK, extracellular signal-regulated kinase; JNK, c-Jun *N*-terminal kinases; NO, nitric oxide; PI3K, phosphatidylinositol 3-kinase; mTORC, mammalian target of rapamycin complex; SAR, structure–activity relationship; NMR, nuclear magnetic resonance; TNFα, tumor necrosis factor α; DMSO, dimethyl sulfoxide; DMAP, 4-dimethylaminopyridine; STATS, signal transducer and activator of transcription proteins; JAK, Janus kinases; COX-2, cyclooxygenase-2; fMLF/CB, N-formyl-methionyl-leucylphenylalanine/cytochalasin B; PDGF, platelet-derived growth factor; EGF, epidermal growth factor; HSP90, heat shock protein 90.
